# Gynecology residents' preparedness to perform standard gynecological procedures autonomously: A national survey among French residents and faculty

**DOI:** 10.1002/ijgo.70068

**Published:** 2025-03-17

**Authors:** Laura Puroski, Sophia Braund, Olivia Guerin, Eric Verspyck, Patrice Crochet, Salma Touleimat

**Affiliations:** ^1^ Department of Obstetrics and Gynaecology, Charles Nicolle Hospital University of Rouen Rouen France; ^2^ Department of Biostatistics and Clinical Research CHU Rouen Rouen France; ^3^ Univ Rouen Normandie, INSERM, NORDIC UMR 1239 ‐ Team Adrenal and Gonadal Pathophysiology (AGoPath) Rouen France

**Keywords:** education, gynecology, surgical techniques, training

## Abstract

**Objective:**

This study aimed to explore the perceptions of French residents and academic teachers regarding residents' autonomy during training and their preparedness to perform standard gynecological procedures.

**Methods:**

A national survey of French obstetrics and gynecology residents and their academic teachers was conducted using an online questionnaire distributed in 2023, which gathered opinions on the level of autonomy granted in the operating room for performing standard gynecological procedures and perceptions of preparedness upon graduation. The survey also examined perceptions on the impact of factors that improve surgical autonomy.

**Results:**

The response rate was 43% (510/1197) for residents and 31% (42/137) for academic teachers. The opinions of residents and academic teachers regarding residents' surgical preparedness by graduation were similar, except for vaginal hysterectomy (30% vs. 57%, *P* < 0.001). Residents were thought prepared to perform hysteroscopy and adnexal surgery by laparoscopy by >90% of respondents. Regarding hysterectomy, opinions varied depending on the approach: the only approach for which more than two‐thirds of residents (67%) were thought to be prepared by gradation was laparoscopy. Regarding factors promoting surgical autonomy, 99% of residents and 97% of teachers considered case volume and coaching quality essential. Only 37% of residents and 55% of teachers deemed the introduction of summative assessment relevant.

**Conclusion:**

French residents and academic teachers reported adequate preparedness for hysteroscopy and adnexal surgery. There were concerns regarding preparedness for more complex surgeries, particularly laparotomic and vaginal hysterectomy. These findings underscore the need for revisions in training methods and residency objectives.

## 1 INTRODUCTION

French residency programs in obstetrics and gynecology (OB‐GYN) rely primarily on the traditional apprenticeship model, with increasing integration of simulation‐based training.^1^ Since 2017, training in OB‐GYN has focused on the acquisition of skills and lasts for 6 years, including the last two, during which the resident must acquire progressive and adapted autonomy. Each graduating resident is expected to achieve “satisfactory competence” in autonomously performing common surgical skills in OB‐GYN. To date, the scope of the surgical procedures to be mastered is implicit but not clearly defined. In addition, there are no recommendations regarding the number of surgical procedures that should be performed autonomously or standardized practical evaluations to be completed before the end of residency.

For many years, barriers to surgical training have been identified across a wide variety of surgical specialties, including gynecology. These barriers include reduced access to supervised operating time during training, increasing diversity of the surgical techniques being performed, a reduction in working hours, and a shift towards medical solutions that reduce surgical volume.^2^, ^3^, ^4^ Furthermore, the dual nature of the specialty makes accessing gynecology experience for trainees comparatively difficult, owing to the competitive nature of an often busy obstetric service.^5^

Consequently, concerns are growing regarding the preparedness level of gynecology residents. A recent Irish study between 2014 and 2024 noted a decline in gynecological surgical confidence, with fewer residents feeling prepared to perform key procedures, such as hysterectomy by laparotomy and vaginal hysterectomy.^6^ According to a recent French survey, only 57% of OB‐GYN residents with a surgical orientation considered their training to be at least adequate.^7^

The main objective of this survey was to assess the opinions of French OB‐GYN residents and teachers concerning the autonomy granted during residency and the level of preparedness obtained at the end of residency for performing standard gynecological surgery procedures. The secondary objective was to find out the opinions of participants on the factors that could improve the autonomy of residents throughout their training.

## 2 MATERIALS AND METHODS

From February to May 2023, all French OB‐GYN residents and all academic teachers were invited to participate in this survey. The participants' responses were anonymized. The questionnaire for residents was distributed through the Association of Obstetricians and Gynecologists in training (AGOF) via personalized email. Academic teachers from every residency program in France were invited via personal email. Two rounds of reminders were issued.

A specific questionnaire was created for residents and academic teachers. Each questionnaire was divided into two parts: the first concerned gynecological surgery and the second concerned obstetrical procedures. Only the first part is presented in this article.

The questionnaire for residents collected demographic data (age, gender, year of residency), wish for future sub‐specialization, city hosting the academic residency program and size of the program (classified as large centers with >30 residents or small centers with <30 residents). The questionnaire focused on “standard” procedures—those relevant for practitioners with a mixed practice in gynecology and obstetrics. Complex procedures requiring advanced specialization, such as those related to oncology, endometriosis, or prolapse, were excluded. Residents were asked whether they were granted autonomy to perform standard gynecological surgery procedures in the operating room (OR) with attending oversight to ensure patient safety. In addition, residents were asked to give their opinions on their expected preparedness to perform standard gynecological procedures independently by the time of graduation. Residents' opinions were collected regarding the importance of being able to perform standard gynecology procedures independently by graduation. The questionnaire also asked residents to assess the usefulness of five factors in improving autonomy: number of cases completed as part of the companionship, quality of coaching, simulation training, formative assessment, and summative assessment.

The questionnaire for academic teachers collected demographic data: age, status, city hosting the residency program, and the main clinical domain of practice (i.e., surgery, obstetrics, mixed). Questions identical to those in the questionnaire for residents were asked: opinions on the preparedness of residents to perform standard procedures independently by graduation, opinions on the importance of being able to perform standard procedures independently by graduation, and opinions on the usefulness of the five factors in improving resident autonomy. Details of the two questionnaires are provided in the Appendix S1.

All questions regarding opinions on preparedness to perform surgical procedures independently were answered on a five‐point Likert scale, ranging from “strongly agree” to “strongly disagree.” The residents who responded with “strongly agree” or “agree” were considered prepared, and those who responded with “neutral,” “disagree” or “strongly disagree” were considered not prepared. Regarding questions on the usefulness of factors promoting autonomy, possible answers were 1 “not at all useful,” 2 “not very useful,” 3 “useful,” or 4 “very useful.” Scores 1 and 2 were considered non‐useful and 3 and 4 were considered useful.

### 2.1 Statistical analyses

Descriptive analyses were performed overall and by year of residency and future sub‐specialization if appropriate. The results are expressed as frequency and percentage (*n*, %) for categorical variables. Dichotomous variables (e.g., prepared/not prepared to perform surgical procedures, useful/non‐useful of factors promoting autonomy) were compared among categories of other variables using Pearson's chi‐squared test. A logistic regression model was used to assess factors independently associated with resident's autonomy during laparoscopic hysterectomy in the context of companionship, and the odds ratios were estimated with a corresponding 95% confidence interval (CI). All statistical tests used a two‐sided 0.05 significance threshold. All statistical analyses were performed using SAS software, version 9.4 (SAS Institute, Cary, NC, USA).

## 3 RESULTS

The response rate was 43% (510/1197) for residents and 31% (42/137) for academic teachers. Residents from all 28 cities hosting a residency program responded, with an average of 15 participants per center. Academic teachers from 25 out of 28 cities hosting a residency program responded, with an average of two participants per center. There were 15 large and 13 small academic centers.

The characteristics of the two groups are detailed in Table 1. Eighty‐three percent (425/510) of residents declared future career plans that include surgical practice in the spectrum of this survey (either by exclusive specialization in surgery or by a mixed practice of gynecology and obstetrics).

**TABLE 1:** Characteristics of residents (*n* = 510) and academic teachers (*n* = 42).

Residents (*n* [%])		Academic teachers (*n* [%])	
Gender		Gender	
Female	437 (86)	Female	9 (21)
Male	73 (14)	Male	33 (79)
Age (years)		Academic status	
<25	40 (8)	Professor (PU‐PH)	37 (88)
25–30	438 (86)	Associate professor (MCU‐PH)	5 (12)
>30	32 (6)		
Year of residency		Years of experience	
1	77 (15)	<5	11 (26)
2	86 (17)	5–10	14 (33)
3	100 (20)	10–15	8 (19)
4	118 (23)	>15	9 (21)
5	74 (14)		
6	55 (11)		
Future career plans		Sub‐specialty	
General obstetrics and gynecology	249 (49)	Surgery	30 (71)
Surgery	176 (35)	Obstetrics	7 (17)
Maternal‐fetal medicine	58 (11)	Obstetrics and surgery	5 (12)
Fertility and reproductive medicine	22 (4)		
Medical gynecology	5 (1)		

The opinions on the importance of being prepared for each standard surgical procedure by graduation are shown in Table 2. More than 75% of respondents from both groups thought that hysteroscopy, adnexal surgery by laparoscopy, and hysterectomy by laparoscopy and laparotomy were important. Residents were less convinced than academic teachers about the importance of being prepared to perform vaginal hysterectomy (41% vs. 76%, *P* < 0.001). Robotic‐assisted hysterectomy was considered important by less than 25% of the respondents.

**TABLE 2:** Resident‐reported importance of surgical procedures for their future career compared with academic teachers' assessment of importance.

	Residents (*n* = 510) (*n* [%])	Academic teachers (*n* = 42) (*n* [%])	*P*
Hysteroscopy procedures	454 (89)	39 (93)	0.439
Adnexal surgery by laparoscopy	497 (97)	40 (95)	0.397
Vaginal hysterectomy	207 (41)	32 (76)	<0.001
Laparoscopic hysterectomy	384 (75)	34 (81)	0.411
Hysterectomy by laparotomy	469 (92)	38 (90)	0.735
Robotic‐assisted hysterectomy	71 (14)	10 (24)	0.082

The opinions regarding residents' surgical preparedness by graduation are presented in Table 3. Opinions were very similar between residents and academic teachers, except for vaginal hysterectomy (30% vs. 57%, *P* < 0.001). Residents were thought to be prepared to perform hysteroscopy and adnexal laparoscopic surgery by more than 90% of respondents. Regarding hysterectomy, opinions varied depending on the approach; the only approach for which more than two‐thirds of residents were thought to be prepared by gradation was laparoscopy (67%).

**TABLE 3:** Resident surgical preparedness by graduation as reported by residents and academic teachers from French obstetrics and gynecology residency programs.

	Residents (*n* = 510) (*n* [%])	Academic teachers (*n* = 42) (*n* [%])	*P*
Hysteroscopy procedures	475 (93)	38 (90)	0.518
Adnexal surgery by laparoscopy	475 (93)	38 (90)	0.518
Vaginal hysterectomy	151 (30)	24 (57)	<0.001
Laparoscopic hysterectomy	351 (67)	28 (67)	0.772
Hysterectomy by laparotomy	304 (60)	25 (60)	0.991
Robotic‐assisted hysterectomy	55 (11)	5 (12)	0.823

**FIGURE 1:**
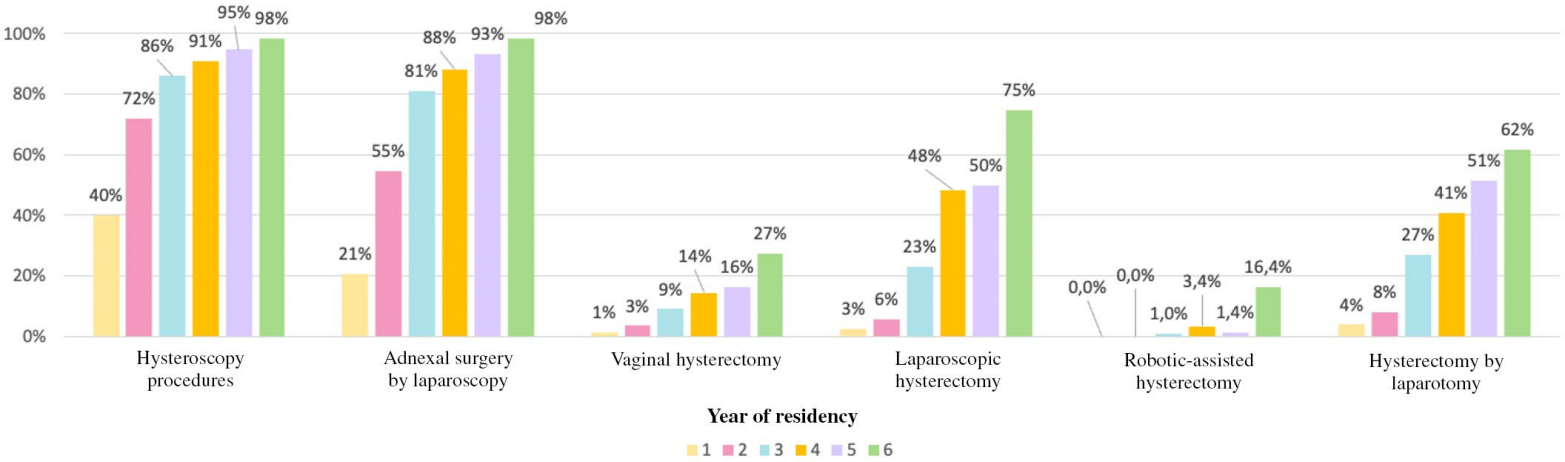
Statement by residents regarding the autonomy granted to the operating room during companionship: Answers to the question: “In practice, are you allowed to perform the following procedures autonomously (with a supervisor present for patient safety)?”.

The statements by residents regarding the autonomy granted in the OR during companionship are presented in Figure 1. Regarding hysteroscopy and adnexal surgery, autonomy occurred for more than 80% of residents by the third year of residency. Regarding hysterectomy, the only approach for which autonomy occurred for more than three‐quarters of residents was laparoscopy. This occurred during their last year of residency.

Multivariate analysis revealed that the year of residency, the size of the program, and the desire for future sub‐specialization in surgery were independently associated with residents' autonomy during laparoscopic hysterectomy in circumstances of companionship (Table 4).

**TABLE 4:** Multivariate analysis of factors associated with resident's autonomy during laparoscopic hysterectomy in a context of companionship.

Odds ratio estimates				
Effect	Point estimate	95% Wald		*P* value
		Confidence limits		
Year of residency	2.483	2.070	2.979	**<0.0001**
Gender				
Female	1			0.9278
Male	1.030	0.543	1.953	
Size of the center				
Small	1			**0.0059**
Large	2.002	1.222	3.280	
Wish for future sub‐specialization				
Mixed practice gynecology and obstetrics	1			**<0.0001**
Gynecology surgery	2.817	1.713	4.633	
Gynecology without surgery	0.674	0.355	1.281	

**FIGURE 2:**
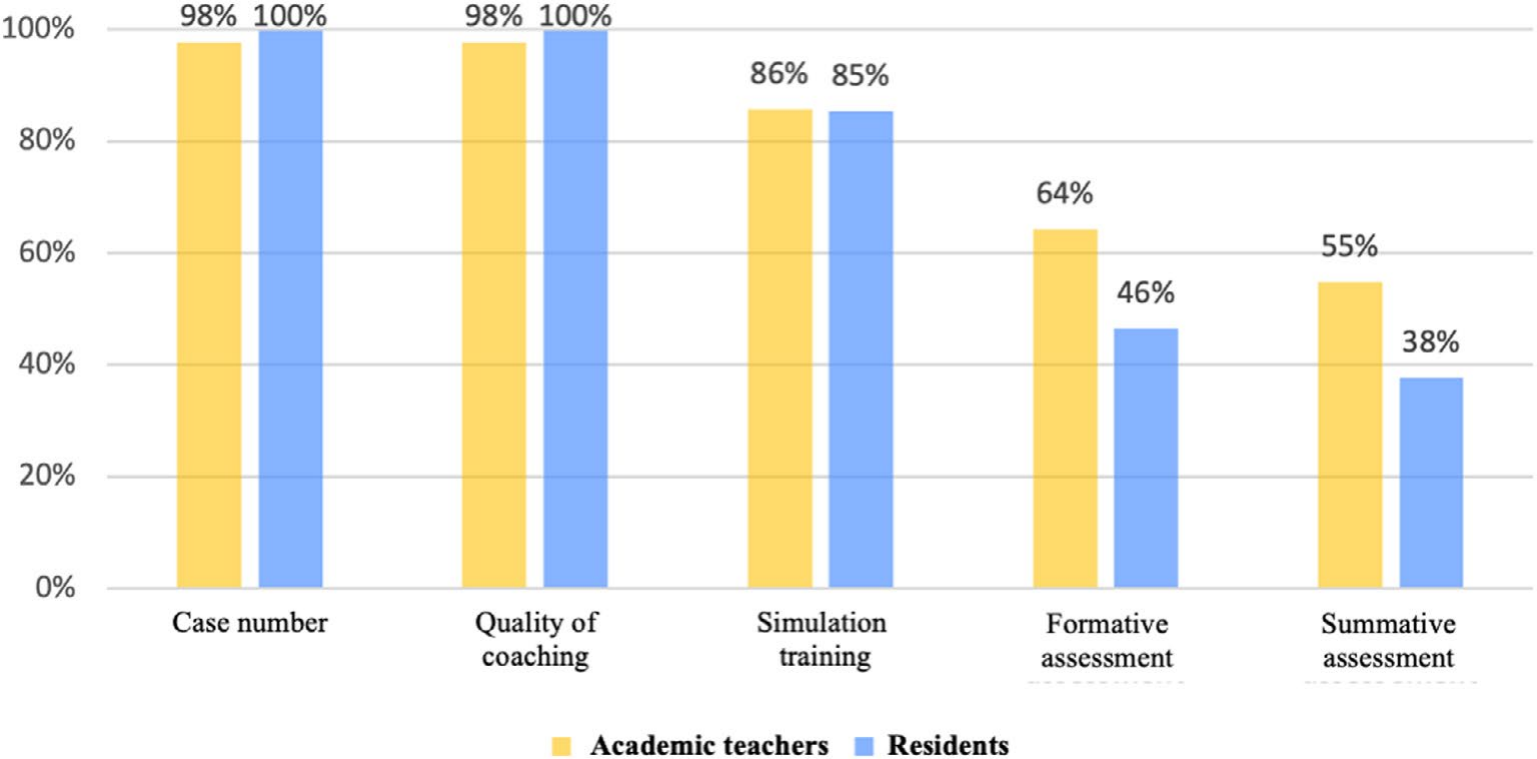
Residents' and academic teachers' opinion on the usefulness of five factors for improving autonomy in the operating room.

Regarding the usefulness of factors for improving autonomy in the OR, the two main factors identified were the number of cases and the quality of coaching. In contrast, formative and summative assessments were considered useful by a minority of residents and academic teachers (Figure 2).

## 4 DISCUSSION

This national survey of French OB‐GYN residents and faculty revealed that degree of preparedness differs depending on the nature of the procedures. Opinions were similar between residents and teachers for the most common questions. Autonomy is given early during residency for operative hysteroscopy and laparoscopic adnexal surgery. Consequently, French gynecology surgeons are thought to be well prepared to perform these procedures by graduation. Conversely, training opportunities, including autonomy with oversight, take place late during residency for all hysterectomies, whatever the approach. The perceived degree of preparedness for hysterectomies varied depending on the approaches. Laparoscopy was clearly the approach for which respondents felt the highest degree of preparedness. Training on hysterectomy by laparotomy is under scrutiny, with a perceived degree of preparedness of 60% only by graduation. A decline in surgical volume is probably part of the explanation.^3^ The decline of the vaginal route is also confirmed, as previously reported in other countries.^8^ Academic teachers underestimated this lack of preparedness compared with residents. Consequently, the teaching of vaginal hysterectomy for all gynecology residents can no longer be considered an achievable goal.^9^ The very low figures regarding robotic‐assisted hysterectomy are likely due to limited access to the robot for residents.^10^

The analysis of factors that could influence the autonomy given to residents for laparoscopic hysterectomy confirms that a wish for future surgical sub‐specialty favors the opportunities experienced by residents. This is a reminder that the apprenticeship model is a dynamic one in which the attitudes and motivations of residents have a strong impact on the quality of their training. However, other factors appear to play a role, such as the size of the training program. This could be explained by a higher surgical volume in larger centers. Surprisingly, gender did not seem to influence the autonomy reported by the residents. These results are not in line with the many studies that concluded that female surgical residents had a lower self‐perception of competence and autonomy than men.^11^, ^12^

A survey by Banks et al. explored the confidence of US OB‐GYN residents and program directors regarding residents' preparedness to perform standard gynecological procedures.^13^ The outcomes were more optimistic than in the present study; more than 90% of respondents thought the residents were able to perform hysterectomies by laparotomy and laparoscopy by graduation. This gap cannot be explained by the length of residency in the United States, which is 2 years less than in France. One explanation could lie in the American training system, which asks for a minimum number of procedures to be completed during the training program, thus fueling the confidence of gynecology trainees in their capacity to be prepared by graduation. The French global reform of residency stated that the fifth and sixth years of residency are dedicated to promoting progressive autonomy to trainees.^1^ However, there is currently no systematic pedagogical framework across French programs to monitor the autonomy experiences of residents.

Taking up the challenges of training for preparedness for surgical practice in the modern environment implies a number of issues to be addressed: skills and knowledge preparation prior to clinical exposure in the OR, a strong and a well structured training environment, and finally an objective measurement of the autonomy given in the OR.

The optimal preparation of residents outside the OR should be driven by competency‐based models, by which validated skills and knowledge assessment tools test residents' achievement of core skills. These curricula are largely based on simulations in surgery.^14^, ^15^ However, simulation has limitations, especially with regard to the transfer of acquired skills to the complex settings of the OR. The concept of entrustable professional activities (EPAs) that are adapted to surgical practice is one of the proposed strategies to bridge the assessment gap between simulation and independent practice.^16^ EPAs are defined as the essential tasks of a profession that require an adequate integration of knowledge, skills, and attitudes from different aspects of performance (e.g., surgical knowledge, communication skills, professionalism). These tasks must be entrusted only to qualified trainees who perform them independently within a time frame, resulting in observable processes and results.^17^

Beyond the assessment of essential surgical tasks by EPAs, the ultimate evaluation of preparedness to perform a given procedure should be objectively measured by the level of autonomy given during a real procedure that is successfully performed with supervisory oversight. The most frequently cited assessment tools for autonomy in the OR are the Zwisch scale and the SIMPL tool.^18^ However, such development implies a culture that encourages the evaluation of residents. The low level of confidence in formative and summative assessments revealed by residents and, more surprisingly, by academic teachers needs to be questioned in future studies.

There is a need to address concerns about the insufficient training opportunities in the OR identified in this survey. One of the answers is to reach consensus on a list of core competencies in OB‐GYN that are necessary for all graduating residents.^19^ Residency tracking based on postgraduate practice goals needs to be further explored. It is likely that not all graduating residents need to be competent in all the listed procedures, especially if they are not going into sub‐specialty practice. It should be possible to limit the number of trainees who seek to achieve proficiency in operative gynecology to focus the training efforts on them. Another response to balance the lack of training opportunities in the OR lies in the enhancement of the preoperative preparation of residents through evidenced‐based simulation curricula and well‐designed learning resources, including videos. Thus, the educational value of the OR experience could be optimized.

The strengths of this survey include the large sample size of residents and academic teachers across all French OB‐GYN residency programs. The range of questions included not only opinions about the alleged surgical preparedness of residents but also residents' experiences about real opportunities experienced during companionship in the OR. This latter point highlights the limited capacity of the educational system to provide training opportunities regarding the prospect of preparing residents for independent practice.

However, the study has limitations. The reliance on self‐reported data introduces the possibility of recall bias, particularly regarding the frequency and autonomy of performing specific procedures. The survey did not assess any objective measures of competence, such as actual surgical outcomes or validated skill assessments, which could provide a more comprehensive view of preparedness. Additionally, the degree of preparedness perceived by respondents must be modulated by the level of complexity of surgical procedures.^20^ Due to the nature of this survey, details about difficult versus complex cases were not specified. However, the level of competence expected by the end of a residency program was implicitly limited to cases of simple‐to‐average complexity, whereas expectations may be higher for a surgical fellowship program.

## 5 CONCLUSION

This national survey of French OB‐GYN residents and faculty revealed areas of strength and weakness within current surgical training programs. While most residents feel adequately prepared for procedures such as hysteroscopy and adnexal surgery via laparoscopy, there are significant concerns regarding their preparedness for more complex surgeries, especially hysterectomy performed by laparotomy or the vaginal approach. These findings suggest the need for revisions of residency training curricula and objectives to ensure that residents are truly prepared for independent practice upon graduation.

## AUTHOR CONTRIBUTIONS

The corresponding author confirms that all authors participated actively and sufficiently in the study. Conception and design of study: Salma Touleimat, Sophia Braund, Patrice Crochet, and Laura Puroski; data collection: Laura Puroski; data analysis and interpretation: Olivia Guerin, Laura Puroski, Eric Verspyck; manuscript preparation: Laura Puroski, Patrice Crochet, Sophia Braund, and Eric Verspyck.

## FUNDING INFORMATION

This study was funded by the Rouen Normandie University.

## CONFLICT OF INTEREST STATEMENT

The authors have no conflicts of interest.

## Supporting information


**Appendix S1.** Questionnaires for residents and academic teachers.

## Data Availability

Data available on request from the authors.
