# Supplementary material for: Gynecology residents' preparedness to perform standard gynecological procedures autonomously: A national survey among French residents and faculty
Source: Int J Gynaecol Obstet. 2025 Mar 17;170(2):875–81. doi: 10.1002/ijgo.70068 (PMC12255914; doi:10.1002/ijgo.70068)
Supplement: Supplementary file 1 — Appendix S1. Questionnaires for residents and academic teachers. [file IJGO-170-875-s001.docx]

1. **Questionnaire to residents :**

*English version* :

**Question n°1** : How old are you ?

**Question n°2** : Are you a female or a male

**Question n°3 :** What is your year of internship?

- 1 to 6

**Question n°4** : In which French centre do you practice?

- Amiens, Angers, Antilles - Guyane, Besançon, Bordeaux, Brest, Caen, Clermont Ferrand, Dijon, Grenoble, La Réunion, Lille, Limoges, Lyon, Marseille, Montpellier-Nîmes, Nancy, Nantes, Nice, Paris, Poitiers, Reims, Rennes, Rouen, Saint Etienne, Strasbourg, Toulouse, Tours.

**Question n°5:** In which area would you like to focus your future practice?

- General obstetrics and gynaecology

- Medically Assisted reproduction

- Antenatal diagnosis

- Gynaecological surgery (oncology, endometriosis, etc.)

- Medical gynaecology

**Question n°6:**

In practice, are you allowed to carry out the following procedures independently (supervisor present for patient safety)?

|  | strongly disagree | disagree | Neutral | agree | strongly agree |
| --- | --- | --- | --- | --- | --- |
| Hysteroscopy procedures |  |  |  |  |  |
| Adnexal surgery by laparoscopy (simple cysts, salpingectomy, annexectomy) |  |  |  |  |  |
| Vaginal hysterectomy |  |  |  |  |  |
| Laparoscopic hysterectomy |  |  |  |  |  |
| Hysterectomy by laparotomy |  |  |  |  |  |
| Robotic-assisted hysterectomy |  |  |  |  |  |

**Question n°7**

Do you think you will have acquired the necessary skills to carry out the following procedures completely independently (without supervision) by the end of your residency ?

|  | strongly disagree | disagree | Neutral | agree | strongly agree |
| --- | --- | --- | --- | --- | --- |
| Hysteroscopy procedures |  |  |  |  |  |
| Adnexal surgery by laparoscopy (simple cysts, salpingectomy, annexectomy) |  |  |  |  |  |
| Vaginal hysterectomy |  |  |  |  |  |
| Laparoscopic hysterectomy |  |  |  |  |  |
| Hysterectomy by laparotomy |  |  |  |  |  |
| Robotic-assisted hysterectomy |  |  |  |  |  |

**Question n°8**

Do you think you will have acquired the necessary skills to carry out the following procedures completely independently (without supervision) by the end of your residency ?

|  | strongly disagree | disagree | Neutral | agree | strongly agree |
| --- | --- | --- | --- | --- | --- |
| Hysteroscopy procedures |  |  |  |  |  |
| Adnexal surgery by laparoscopy (simple cysts, salpingectomy, annexectomy) |  |  |  |  |  |
| Vaginal hysterectomy |  |  |  |  |  |
| Laparoscopic hysterectomy |  |  |  |  |  |
| Hysterectomy by laparotomy |  |  |  |  |  |
| Robotic-assisted hysterectomy |  |  |  |  |  |

**Question n°9**

The following elements help to promote autonomy when carrying out procedures. Can you rate their usefulness on a scale of 1 (not very useful) to 4 (very useful) for surgery ?

|  | 1  Not very useful | 2  Moderately useful | 3  Useful | 4  Very useful |
| --- | --- | --- | --- | --- |
| Case number during compagnonship |  |  |  |  |
| The quality of coaching |  |  |  |  |
| Simulation training |  |  |  |  |
| Formative assessement, with outcomes shared to other team members |  |  |  |  |
| Summative assessement, with outcomes shared to other team members |  |  |  |  |

**Question n°10:** open comments

*French version*

**Question n°1** : Quel âge avez-vous ?

**Question n°2** : Êtes-vous :

-       Un homme

-       Une femme

**Question n°3** : Quel est votre année d’internat ?

-       1 à 6

**Question n°4** : Dans quelle subdivision exercez-vous ?

-     Amiens, Angers, Antilles – Guyane, Besançon, Bordeaux, Brest, Caen, Clermont Ferrand, Dijon, Grenoble, La Réunion, Lille, Limoges, Lyon, Marseille, Montpellier-Nîmes, Nancy, Nantes, Nice, Paris, Poitiers, Reims, Rennes, Rouen, Saint Etienne, Strasbourg, Toulouse, Tours.

**Question n°5** : Dans quel domaine souhaitez-vous orienter votre pratique future ?

-       Gynécologie-obstétrique généraliste

-       Aide Médicale à la Procréation

-       Diagnostic Anténatal

-       Chirurgie gynécologique (oncologie, endométriose, ..)

-       Gynécologie médicale

**Question n°6 :**

En pratique, vous laisse-t-on réaliser en autonomie les interventions suivantes (superviseur présent pour la sécurité de la patiente) ?

|  | Pas du tout d’accord | Pas d’accord | Neutre | D’accord | Totalement d’accord |
| --- | --- | --- | --- | --- | --- |
| une hystéroscopie opératoire |  |  |  |  |  |
| une chirurgie annexielle (kyste simple, salpingectomie, annexectomie) par coelioscopie |  |  |  |  |  |
| une hystérectomie par voie basse |  |  |  |  |  |
| une hystérectomie coelioscopique |  |  |  |  |  |
| une hystérectomie au robot |  |  |  |  |  |
| une hystérectomie par laparotomie |  |  |  |  |  |

**Question n°7**

Pensez-vous que vous aurez acquis les compétences nécessaires à la réalisation des procédures suivantes en autonomie totale (sans supervision) à l'issue de votre internat ?

|  | Pas du tout d’accord | Pas d’accord | Neutre | D’accord | Totalement d’accord |
| --- | --- | --- | --- | --- | --- |
| une hystéroscopie opératoire |  |  |  |  |  |
| une chirurgie annexielle (kyste simple, salpingectomie, annexectomie) par coelioscopie |  |  |  |  |  |
| une hystérectomie par voie basse |  |  |  |  |  |
| une hystérectomie coelioscopique |  |  |  |  |  |
| une hystérectomie au robot |  |  |  |  |  |
| une hystérectomie par laparotomie |  |  |  |  |  |

**Question n°8**

Les compétences pour réaliser les procédures suivantes sont-elles importante à acquérir pour la pratique future des praticiens diplômés de gynécologie obstétrique ?

|  | Pas du tout d’accord | Pas d’accord | Neutre | D’accord | Totalement d’accord |
| --- | --- | --- | --- | --- | --- |
| une hystéroscopie opératoire |  |  |  |  |  |
| une chirurgie annexielle (kyste simple, salpingectomie, annexectomie) par coelioscopie |  |  |  |  |  |
| une hystérectomie par voie basse |  |  |  |  |  |
| une hystérectomie coelioscopique |  |  |  |  |  |
| une hystérectomie au robot |  |  |  |  |  |
| une hystérectomie par laparotomie |  |  |  |  |  |

**Question n°9**

Les éléments suivants permettent de favoriser l'autonomie pour réaliser des gestes. Pouvez-vous évaluer leur utilité sur une échelle de 1 (peu utile) à 4 (très utile) pour la chirurgie ?

|  | 1  (très peu utile) | 2 | 3 | 4  (très utile) |
| --- | --- | --- | --- | --- |
| Le nombre de cas réalisés en compagnonnage |  |  |  |  |
| La qualité du coaching de l'enseignant pendant le compagnonnage |  |  |  |  |
| Les séances de simulation |  |  |  |  |
| Une évaluation formative dont les résultats sont communiqués aux autres membres de l'équipe enseignante |  |  |  |  |
| Une évaluation sommative (= certification) dont les résultats sont communiqués aux autres membres de l'équipe enseignante |  |  |  |  |

**Question n°10**

Commentaires libres :

1. **Questionnaire to academic teachers:**

*English version*

**Question n°1:** Are you a female or a male ?

**Question n°2** : How many years have you been a professor or assistant-professor ?

- 5 years

- 5 - 10 years

- 10 - 15 years

- > 15 years

**Question n°3** : In which centre do you practice?

Amiens, Angers, Antilles - Guyane, Besançon, Bordeaux, Brest, Caen, Clermont Ferrand, Dijon, Grenoble, La Réunion, Lille, Limoges, Lyon, Marseille, Montpellier-Nîmes, Nancy, Nantes, Nice, Paris, Poitiers, Reims, Rennes, Rouen, Saint Etienne, Strasbourg, Toulouse, Tours.

**Question n°4**: Do your activities (clinical-teaching-research) focus on a particular area of specialisation?

- Obstetrics

- Surgery

- No sub-specialisation

**Question n°5:**

Upon graduation, I believe that resident are fully capable of performing the following procedures independently (without supervision)

|  | strongly disagree | disagree | Neutral | agree | strongly agree |
| --- | --- | --- | --- | --- | --- |
| Hysteroscopy procedures |  |  |  |  |  |
| Adnexal surgery by laparoscopy (simple cysts, salpingectomy, annexectomy) |  |  |  |  |  |
| Vaginal hysterectomy |  |  |  |  |  |
| Laparoscopic hysterectomy |  |  |  |  |  |
| Hysterectomy by laparotomy |  |  |  |  |  |
| Robotic-assisted hysterectomy |  |  |  |  |  |

**Question n°6:**

Are the skills needed to carry out the following procedures essential for the future practice of qualified obstetric gynaecologists?

|  | strongly disagree | disagree | Neutral | agree | strongly agree |
| --- | --- | --- | --- | --- | --- |
| Hysteroscopy procedures |  |  |  |  |  |
| Adnexal surgery by laparoscopy (simple cysts, salpingectomy, annexectomy) |  |  |  |  |  |
| Vaginal hysterectomy |  |  |  |  |  |
| Laparoscopic hysterectomy |  |  |  |  |  |
| Hysterectomy by laparotomy |  |  |  |  |  |
| Robotic-assisted hysterectomy |  |  |  |  |  |

**Question n°7** The following elements help to promote autonomy when carrying out procedures. Can you rate their usefulness on a scale of 1 (not very useful) to 4 (very useful) for surgery ?

|  | 1  Not very useful | 2  Moderately useful | 3  Useful | 4  Very useful |
| --- | --- | --- | --- | --- |
| Case number during compagnonship |  |  |  |  |
| The quality of coaching |  |  |  |  |
| Simulation training |  |  |  |  |
| Formative assessement, with outcomes shared to other team members |  |  |  |  |
| Summative assessement, with outcomes shared to other team members |  |  |  |  |

**Question n°8** Open comments

*French version*

**Question n°1** : Êtes-vous :

-       Un homme

-       Une femme

**Question n°2** : Depuis combien d’année(s) êtes-vous PU-PH / MCU-PH ?

- 5 ans
- 5 – 10 ans
- 10 – 15 ans
- > 15 ans

**Question n°3** : Dans quelle ville exercez-vous ?

Amiens, Angers, Antilles – Guyane, Besançon, Bordeaux, Brest, Caen, Clermont Ferrand, Dijon, Grenoble, La Réunion, Lille, Limoges, Lyon, Marseille, Montpellier-Nîmes, Nancy, Nantes, Nice, Paris, Poitiers, Reims, Rennes, Rouen, Saint Etienne, Strasbourg, Toulouse, Tours.

**Question n°4 :** Êtes-vous orienté vers un domaine particulier de la spécialité dans vos activités (clinique-enseignement-recherche) ?

- Obstétrique
- Chirurgie
- Aucune orientation professionnelle

**Question n°5** :

À l'issue de leur internat, je pense qu'ils sont tout à fait capables de pratiquer les procédures suivantes de manière autonome (sans supervision) :

|  | Pas du tout d’accord | Pas d’accord | Neutre | D’accord | Totalement d’accord |
| --- | --- | --- | --- | --- | --- |
| une hystéroscopie opération |  |  |  |  |  |
| une chirurgie annexielle (kyste simple, salpingectomie, annexectomie) par coelioscopie |  |  |  |  |  |
| une hystérectomie par voie basse |  |  |  |  |  |
| une hystérectomie coelioscopique |  |  |  |  |  |
| une hystérectomie au robot |  |  |  |  |  |
| une hystérectomie par laparotomie |  |  |  |  |  |

**Question n°6** :

Les compétences pour réaliser les procédures suivantes sont-elles indispensables à acquérir pour la pratique future des praticiens diplômés de gynécologie obstétrique ?

|  | Pas du tout d’accord | Pas d’accord | Neutre | D’accord | Totalement d’accord |
| --- | --- | --- | --- | --- | --- |
| une hystéroscopie opération |  |  |  |  |  |
| une chirurgie annexielle (kyste simple, salpingectomie, annexectomie) par coelioscopie |  |  |  |  |  |
| une hystérectomie par voie basse |  |  |  |  |  |
| une hystérectomie coelioscopique |  |  |  |  |  |
| une hystérectomie au robot |  |  |  |  |  |
| une hystérectomie par laparotomie |  |  |  |  |  |

**Question n°7** :

Les éléments suivants permettent de favoriser l'autonomie pour réaliser des gestes. Pouvez-vous évaluer leur utilité sur une échelle de 1 (très peu utile) à 4 (très utile) pour la chirurgie ?

|  | 1  (très peu utile) | 2 | 3 | 4  (très utile) |
| --- | --- | --- | --- | --- |
| Le nombre de cas réalisés en compagnonnage |  |  |  |  |
| La qualité du coaching de l'enseignant pendant le compagnonnage |  |  |  |  |
| Les séances de simulation |  |  |  |  |
| Une évaluation formative dont les résultats sont communiqués aux autres membres de l'équipe enseignante |  |  |  |  |
| Une évaluation sommative (= certification) dont les résultats sont communiqués aux autres membres de l'équipe enseignante |  |  |  |  |

**Question n°8**

Commentaires libres :
